# Supplementary material for: Discovery and Characterization of an ALFA-Tag-Specific Affinity Resin Optimized for Protein Purification at Low Temperatures in Physiological Buffer
Source: Biomolecules. 2021 Feb 12;11(2):269. doi: 10.3390/biom11020269 (PMC7918568; doi:10.3390/biom11020269)
Supplement: Supplementary file 1 [file biomolecules-11-00269-s001.pdf]

Supplementary Information

Discovery and characterization of an ALFA-tag specific affinity resin optimized for protein purification at low temperatures in physiological buffer.

Kilisch et al.

Supplementary Figures

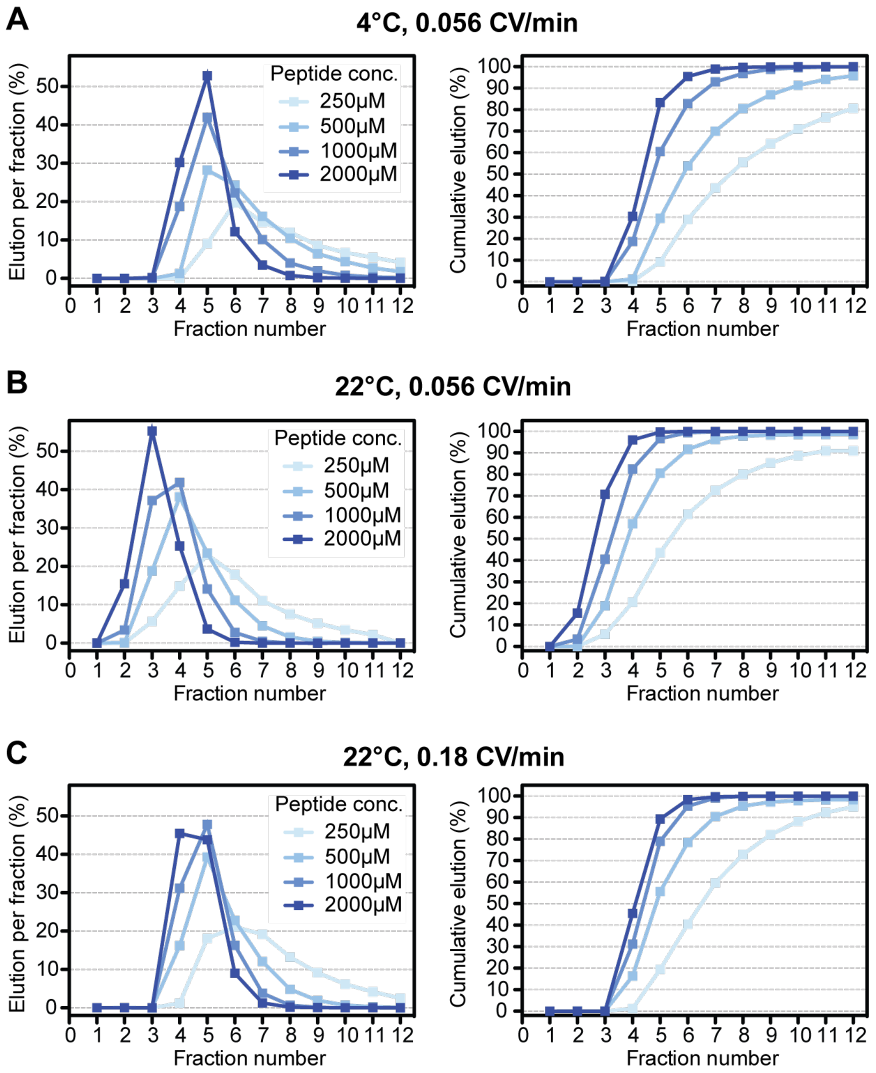

Figure S1: Titration of ALFA peptide concentration. ALFA Selector^{CE} was saturated with shGFP2-ALFA. Columns were eluted in stopped-flow mode at 4°C or 22°C with elution buffer containing different concentrations of ALFA peptide. To achieve different effective flow rates, the time between individual additions of elution buffer aliquots was varied. Eluted protein was quantified by fluorescence. **A**: Stop-flow elution of target protein at 4°C at low effective flow rate of 0.56 CV/min. Peptide concentration used: 250 µM, 500 µM, 1000 µM, 2000 µM. Graphs illustrate the degree of elution achieved per fraction (left panel) and as a cumulative plot (right panel). **B, C**: Stopped-flow elution of target protein at 22°C, at effective flow rates of 0.056 CV/min (**B**), or 0.18 CV/min (**C**).

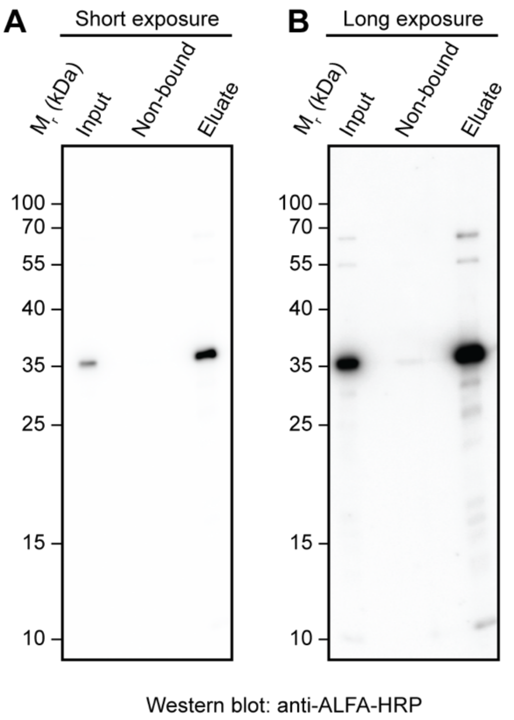

Figure S2, related to Figure 3C. One-step affinity purification of low abundant proteins. 50 mL of HeLa lysate containing 100 nM ALFA-shGFP2 was passed over 1 mL ALFA Selector^{CE} at room temperature using gravity flow. After washing, the column was eluted with PBS containing 1 mM ALFA peptide. Fractions were pooled and analyzed by SDS-PAGE (Figure 3A, B) and Western blotting (shown here and Figure 3C). Amounts loaded correspond to 1/20000 of the input and flow-through material and 1/2000 of the eluate. Shown is a representative blot after short (**A**) and long exposure (**B**). The data shown recapitulates data presented in Figure 3C, here, however, full blots are show.

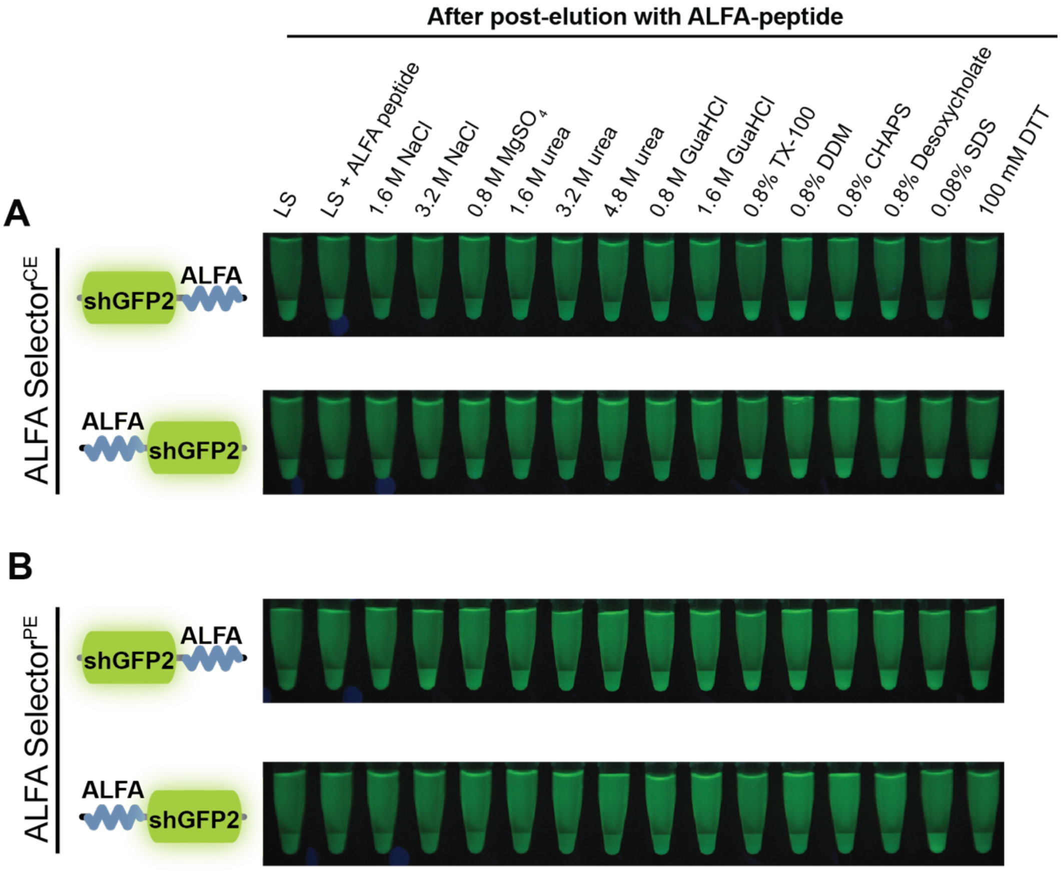

Figure S3, related to Figure 4: Buffer compatibility. ALFA Selectors were saturated with shGFP2-ALFA, or ALFA-shGFP2, washed extensively with PBS and incubated in a 10-fold volume of the indicated substances for 2 h at 22°C. The leakage of target protein from the Selector resin was analyzed by quantifying the fluorescence released into the supernatant before (see Figure 4) and after post elution with ALFA peptide (shown here). The experiment was performed with ALFA Selector^{CE} (upper panel; **A**) and ALFA Selector^{PE} (lower panel, **B**).

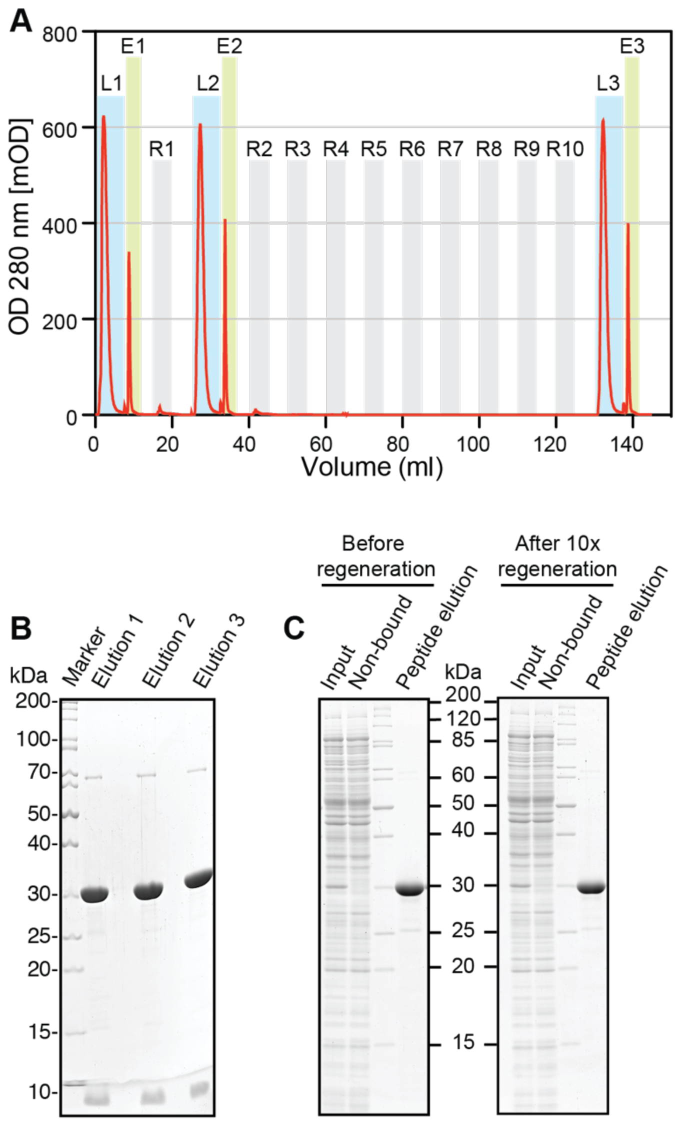

Figure S4, related to Figure 5: Regeneration of ALFA Selector^{CE} under basic conditions. **A**: 0.5 mL of ALFA Selector^{CE} was subjected to a first cycle of loading with ALFA-shGFP2 (L1) and competitive peptide elution (E1). The column was regenerated under basic conditions using 100 mM NaOH (R1) and re-equilibrated with PBS before starting a second cycle of loading and elution (L2 and E2). After 9 additional repeated regeneration/re-equilibration steps (R2-R10), the column was loaded and eluted a third time (L3, E3). The whole procedure was followed by recording the optical density at 280 nm (OD_{280}; red curve). **B**: Fractions of each elution step were collected, quantified and analyzed by SDS-PAGE and Coomassie staining. **C**: Effect of regeneration on non-specific background binding. Single-step affinity purification from *E. coli* lysate blended with shGFP2-ALFA was performed using ALFA Selector^{CE} either before regeneration or after 10 cycles of regeneration with 100 mM NaOH (left and right panel, respectively). The experiment was essentially performed as described for Figures 3A and 3B.

Supplementary Table 1: Plasmids used in this study

Bacterial Expression	Encoded protein	Origin/Citation
pNT1177	ALFA-shGFP2-His_6	Götzke et al. [15]
pNT1050	His_{14}-bdSUMO-shGFP2-ALFA	Götzke et al. [15]
pNT1626	His_{14}-bdSUMO-NbALFA_CE_C1_Syn-Sp-Cys	This study

Supplementary Table 2: Antibodies and Selectors

Antibody/Selector	Supplier	Order No
ALFA Selector^{PE}	NanoTag Biotechnologies	N1510
ALFA SelectorST	NanoTag Biotechnologies	N1511
ALFA Selector^{CE}	NanoTag Biotechnologies	N1512
FluoTag® X2 anti-ALFA HRP	NanoTag Biotechnologies	N1501-HRP

Supplementary Table 3: Primers

Name	Sequence	Reference
CaLl 01	GTC CTG GCT GCT CTT CTA CAA GG	Olichon et al. [26]
CaLl 02	GGT ACG TGC TGT TGA ACT GTT CC	Olichon et al. [26]
F1	TCT GGT GAT GCA TCT GAC AGC GAG GTG CAG CTG CAG GAG TCT GG	Götzke et al. [15]
R1	GTT TTC CCC AGT GGA TCC AGA AGT TTG TGG TTT TGG TGT CTT GGG	Götzke et al. [15]
